# IL-24 Negatively Regulates Keratinocyte Differentiation Induced by Tapinarof, an Aryl Hydrocarbon Receptor Modulator: Implication in the Treatment of Atopic Dermatitis

**DOI:** 10.3390/ijms21249412

**Published:** 2020-12-10

**Authors:** Yen Hai Vu, Akiko Hashimoto-Hachiya, Masaki Takemura, Ayako Yumine, Yasutaka Mitamura, Takeshi Nakahara, Masutaka Furue, Gaku Tsuji

**Affiliations:** 1 Department of Dermatology, Graduate School of Medical Sciences, Kyushu University, Maidashi 3-1-1, Higashiku, Fukuoka 812-8582, Japan; yenvuhai@dermatol.med.kyushu-u.ac.jp (Y.H.V.); ahachi@dermatol.med.kyushu-u.ac.jp (A.H.-H.); take0917@dermatol.med.kyushu-u.ac.jp (M.T.); a-takai@dermatol.med.kyushu-u.ac.jp (A.Y.); mitamura@dermatol.med.kyushu-u.ac.jp (Y.M.); nakahara@dermatol.med.kyushu-u.ac.jp (T.N.); furue@dermatol.med.kyushu-u.ac.jp (M.F.); 2 Division of Skin Surface Sensing, Graduate School of Medical Sciences, Kyushu University, Maidashi 3-1-1, Higashiku, Fukuoka 812-8582, Japan; 3 Research and Clinical Center for Yusho and Dioxin, Kyushu University, Maidashi 3-1-1, Higashiku, Fukuoka 812-8582, Japan

**Keywords:** atopic dermatitis, aryl hydrocarbon receptor, tapinarof, JAK inhibitor, IL-24, filaggrin, loricrin

## Abstract

Skin barrier dysfunction, including reduced filaggrin (FLG) and loricrin (LOR) expression, plays a critical role in atopic dermatitis (AD) development. Since aryl hydrocarbon receptor (AHR), a ligand-activated transcription factor, mediates keratinocyte differentiation, it is a potential target for AD treatment. Recently, clinical studies have shown that tapinarof, an AHR modulator, attenuated the development of AD. To examine the molecular mechanism involved in this, we analyzed tapinarof-treated normal human epidermal keratinocytes (NHEKs). Tapinarof upregulated FLG and LOR mRNA and protein expression in an AHR-dependent manner. Tapinarof also induced the secretion of IL-24, a cytokine that activates Janus kinase (JAK)-signal transducer and activator of transcription (STAT), leading to the downregulation of FLG and LOR expression. Knockdown of either IL-24 or STAT3 expression by small interfering RNA (siRNA) transfection augmented the upregulation of FLG and LOR expression induced by tapinarof, suggesting that inhibition of the IL-24/STAT3 axis during AHR activation supports the improvement of skin barrier dysfunction. Furthermore, tapinarof alone could restore the downregulation of FLG and LOR expression induced by IL-4, a key cytokine of AD, and its combination with JAK inhibitors enhanced this effect. These findings provide a new strategy for treating AD using AHR modulators and JAK inhibitors.

## 1. Introduction

Atopic dermatitis (AD) is a very common, chronic inflammatory skin disease [1]. Since skin barrier defects facilitate allergen priming, leading to allergic responses, improving skin barrier dysfunction is vital in treating AD [2,3]. Our previous studies reported that activation of aryl hydrocarbon receptor (AHR), a ligand-activated transcription factor, upregulates the expression of filaggrin (FLG) and loricrin (LOR), which are essential proteins in skin barrier functions [4,5,6]. Since reduced FLG and LOR expression contributes to the development of AD [3], restoring skin barrier functions via AHR activation is beneficial for treating AD. Previous studies, including ours, have reported that AHR ligands, including coal tar, soybean tar, and 6-formylindolo[3,2-b]carbazole (FICZ), could improve skin barrier dysfunction in AD [7,8,9,10], suggesting that an AHR ligand could become a therapeutic agent for treating AD. Recently, tapinarof, which activates AHR and upregulates CYP1A1 [11], has been shown to attenuate the disease activity of AD clinically [12]. Although it has been demonstrated that tapinarof activates the AHR-NRF2 axis [13], leading to the inhibition of inflammation [11], whether tapinarof modulates skin barrier dysfunction in AD has remained unclear.

To investigate this, we analyzed tapinarof-treated normal human epidermal keratinocytes (NHEKs). We found that tapinarof treatment upregulated FLG and LOR expression; it also upregulated IL-24 expression. IL-24 is included in the IL-10 family of cytokines, and it signals through two heterodimeric receptors: IL-20R1/IL-20R2 and IL-22R1/IL-20R2. Upon binding to its receptors, IL-24 induces activation of the STAT1 and STAT3 transcription factors [14]. It has been shown that IL-24 downregulates FLG and LOR expression via STAT3 in human keratinocytes, indicating that activation of the IL-24/STAT3 axis reduces FLG and LOR expression in AD [15]. Therefore, we assumed that inhibition of the IL-24-STAT3 axis during AHR activation might support the improvement of the skin barrier dysfunction in AD.

Recently, oral or topical JAK inhibitors have been developed for the treatment of AD [16]. Therefore, there is a need to understand which combination of JAK inhibitors with other agents, including tapinarof, is optimal; however, few basic studies on this issue have been performed. JAK inhibitors, such as baricitinib (oral) and JTE-052 (topical), have reportedly achieved clinical improvement in AD [17,18]. Since they can inhibit JAK-STAT signaling activity, we hypothesized that they might modify the capacity of tapinarof to restore skin barrier functions in AD.

Herein, we demonstrate that the combination of tapinarof with JAK inhibitors elicits a synergic effect on improving skin barrier dysfunction in AD.

## 2. Results

### 2.1. Tapinarof Upregulated FLG and LOR Expression via AHR Activation in NHEKs

**Figure 1:**
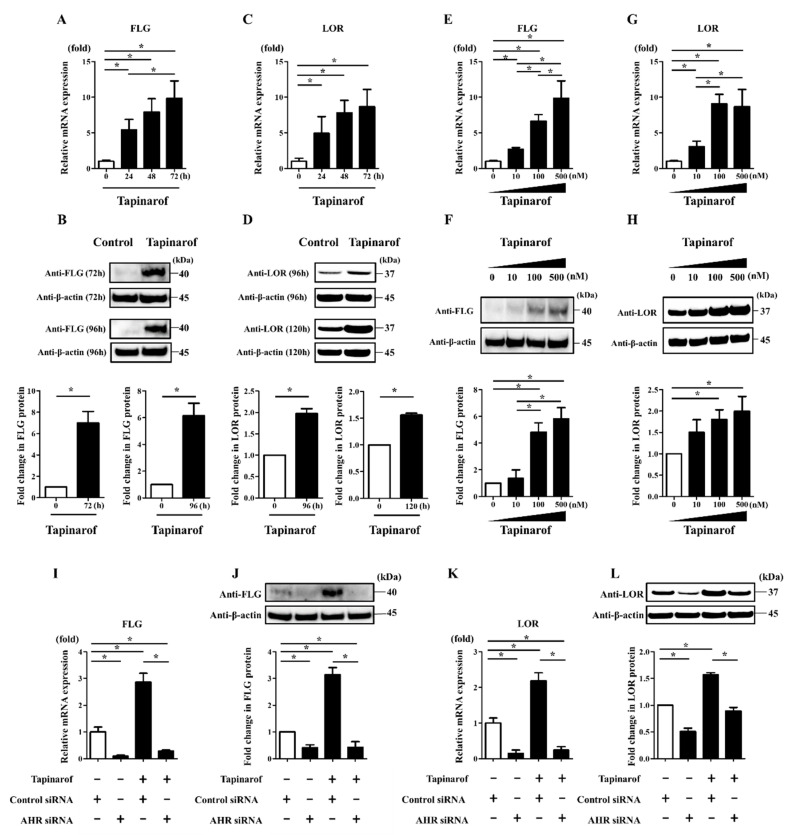
Tapinarof upregulated FLG and LOR expression via AHR activation in NHEKs. (**A**–**D**) NHEKs were treated with tapinarof (500 nM) for the indicated period. (**E**–**H**) NHEKs were treated with tapinarof at the indicated dose, (**I**–**L**) si-control- and si-AHR-transfected NHEKs were treated with tapinarof (500 nM) for 72 h for qRT-PCR and for 96 h for Western blotting analyses. (**A**,**C**,**E**,**G**,**I**,**K**) FLG and LOR mRNA expression was assessed by qRT-PCR. Representative data of three independent experiments with similar results are shown. (**B**,**D**,**F**,**H**,**J**,**L**) FLG and LOR protein expression was analyzed by Western blotting with anti-FLG and anti-LOR antibodies. (**Upper**) Representative blot images of three independent experiments with similar results are shown. (**Lower**) FLG or LOR protein levels are normalized to β-actin protein levels using ImageJ and expressed as fold change over the control group. (**A**–**L**) Error bars represent mean ± standard deviation; *n* = 3 for each group. Statistically significant differences are presented: * *p* < 0.05.

To investigate how the expression of FLG and LOR was mediated by AHR activation, we examined their expression in NHEKs treated with tapinarof. NHEKs were treated with DMSO (control) or tapinarof (500 nM) for 24, 48, and 72 h for qRT-PCR and for 72, 96, and 120 h for Western blotting analyses. Tapinarof upregulated FLG and LOR mRNA (Figure 1A,C) and protein (Figure 1B,D). Furthermore, NHEKs were treated with tapinarof at different doses (10, 100, and 500 nM). Tapinarof upregulated FLG and LOR mRNA in a dose-dependent manner (Figure 1E,G). Western blotting analysis confirmed that tapinarof upregulated FLG and LOR protein in NHEKs (Figure 1F,H). Meanwhile, tapinarof (10, 100, and 500 nM) did not affect cell viability (Supplementary Figure S1).

To further examine whether the effect of tapinarof on the upregulation of FLG and LOR expression is dependent on AHR, NHEKs were transfected with either scrambled siRNA (si-control) or siRNA against AHR (si-AHR). siRNA transfection efficiently knocked down AHR mRNA and protein expression (Supplementary Figure S2A,B). Then, si-control- or si-AHR-transfected NHEKs were treated with tapinarof (500 nM) for qRT-PCR and Western blotting analyses of FLG and LOR expression. Knockdown of AHR canceled this effect on tapinarof-induced upregulation of FLG and LOR mRNA (Figure 1I,K) and protein (Figure 1J,L) in NHEKs. These findings are consistent with our previous reports regarding AHR activation-induced upregulation of FLG and LOR expression in NHEKs [8,9,10,19].

### 2.2. Tapinarof Upregulated IL-24 Expression Partially via AHR Activation in NHEKs

We and other researchers have previously shown that IL-24 is a target cytokine of some oxidative environmental AHR agonists, such as TCDD, benzo(a)pyrene, particulate matter, and UVB irradiation [20,21,22,23]. However, no data have been presented regarding whether IL-24 is produced by keratinocytes treated with tapinarof. Furthermore, our recent study has demonstrated that IL-24 downregulated mRNA expression of FLG and LOR, but not involucrin (IVL), in NHEKs [15]. These findings prompted us to test the possible role of IL-24 in tapinarof-induced upregulation of FLG and LOR expression in keratinocytes.

**Figure 2:**
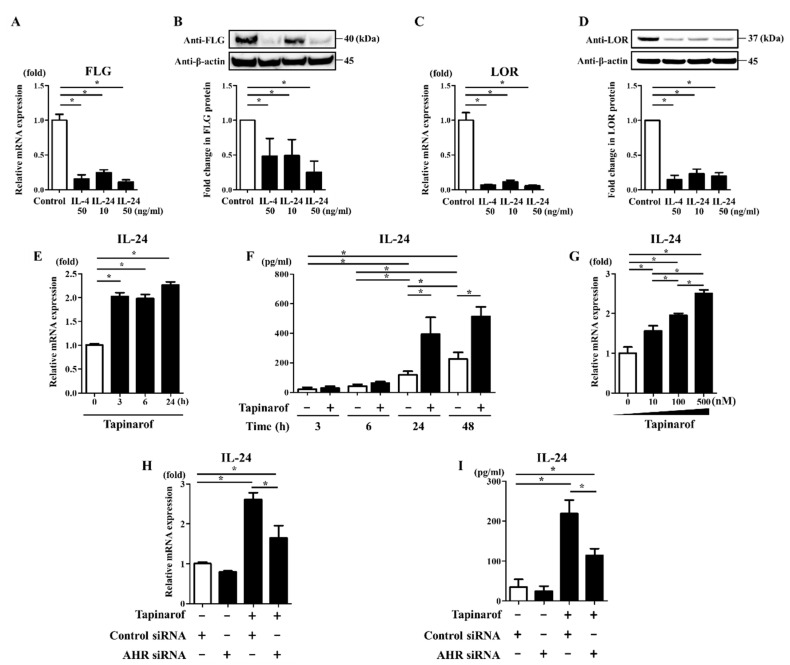
Tapinarof upregulated IL-24 expression via AHR activation in NHEKs. (**A**–**D**) NHEKs were treated with or without IL-4 (50 ng/mL) and IL-24 (10 and 50 ng/mL) for 72 h. (**A**,**C**) FLG and LOR mRNA expression was assessed by qRT-PCR. Representative data of three independent experiments with similar results are shown. (**B**,**D**) FLG and LOR protein expression was analyzed by Western blotting with anti-FLG and anti-LOR antibodies. (**Upper**) Representative blot images of three independent experiments with similar results are shown. (**Lower**) FLG or LOR protein levels are normalized to β-actin protein levels using ImageJ and expressed as fold change over the control group. (**E**,**F**) NHEKs were stimulated with tapinarof (500 nM) for the indicated period. (**G**) NHEKs were stimulated with tapinarof at the indicated dose for 24 h. (**H**,**I**) si-control- and si-AHR-transfected NHEKs were treated with tapinarof (500 nM) for 24 h. (**E**,**G**,**H**) IL-24 mRNA expression was analyzed by qRT-PCR. (**F**,**I**) IL-24 secretion in the culture supernatant was measured using ELISA. Representative data of three independent experiments with similar results are shown. (**A**–**I**) Error bars represent mean ± standard deviation; *n* = 3 for each group. Statistically significant differences are presented: * *p* < 0.05.

First, we re-examined whether IL-24 affects FLG and LOR expression in NHEKs. NHEKs were exposed to IL-4 (50 ng/mL) and IL-24 (10 and 50 ng/mL) for 72 h for qRT-PCR and Western blotting analyses of FLG and LOR expression. We confirmed that IL-4 and IL-24 downregulated FLG and LOR mRNA (Figure 2A,C) and protein (Figure 2B,D).

Next, we examined whether tapinarof upregulates IL-24 expression. For this purpose, NHEKs were treated with DMSO (control) or tapinarof (500 nM) for the indicated period for qRT-PCR of IL-24 mRNA and ELISA of IL-24 secretion in the culture supernatant. Tapinarof upregulated IL-24 mRNA (Figure 2E) and secretion (Figure 2F). We also found that IL-24 was produced in NHEKs, even in unstimulated conditions (Figure 2F). In addition, NHEKs were treated with tapinarof at different doses (10, 100, and 500 nM) for 24 h. Tapinarof upregulated IL-24 mRNA in a dose-dependent manner (Figure 2G). To further examine whether the effect of tapinarof on the upregulation of IL-24 expression is dependent on AHR, si-control- and si-AHR-transfected NHEKs were treated with tapinarof (500 nM) for 24 h for qRT-PCR and ELISA of IL-24 mRNA and secretion. The knockdown of AHR significantly reduced this effect on tapinarof-induced upregulation of IL-24 mRNA (Figure 2H) and secretion (Figure 2I) in NHEKs, suggesting that AHR activation contributes to tapinarof-induced upregulation of IL-24 expression.

### 2.3. Inhibition of IL-24 and STAT3 Enhanced Tapinarof-Induced Upregulation of FLG and LOR Expression in NHEKs

It was previously shown that in keratinocytes, IL-24 binds to its receptor leading to specific activation of JAK/STAT3 without activating other STAT proteins there [14]. IL-24 was also shown to downregulate FLG expression via STAT3, but not STAT6, in human keratinocytes [15]. In addition, activation of STAT3 in keratinocytes was reported to play a critical role in skin allergic inflammation, including downregulation of barrier proteins such as filaggrin and loricrin [24,25]. Based on these findings and the present data showing that tapinarof increased FLG and LOR in NHEKs (Figure 1), and that tapinarof induced the secretion of IL-24 (Figure 2), it is possible that the inhibition of the JAK/STAT3 axis activated by IL-24 might contribute to the upregulation of FLG and LOR expression induced by treatment with tapinarof.

**Figure 3:**
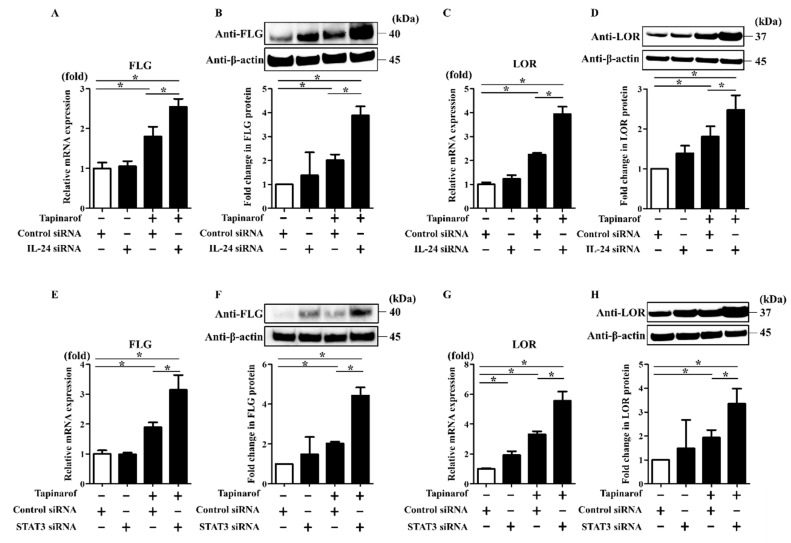
Inhibition of IL-24 and STAT3 enhanced tapinarof-induced upregulation of FLG and LOR expression in NHEKs. NHEKs were transfected with control siRNA (si-control) and siRNA against IL-24 (si-IL-24) (**A**–**D**) or STAT-3 (si-STAT3) (**E**–**H**) and subsequently treated with tapinarof (500 nM) for 96 h. (**A**,**C**,**E**,**G**) FLG and LOR mRNA levels were assessed by qRT-PCR. Representative data of three independent experiments with similar results are shown. (**B**,**D**,**F**,**H**) FLG and LOR protein levels were analyzed by Western blotting. (**Upper**) Representative blot images of three independent experiments with similar results are shown. (**Lower**) FLG or LOR protein levels are normalized to β-actin protein levels using ImageJ and expressed as fold change over the control group. (**A**–**H**) Error bars represent mean ± standard deviation; *n* = 3 for each group. Statistically significant differences are presented: * *p* < 0.05.

To test this, NHEKs were transfected with scrambled siRNA (si-control), siRNA against IL-24 (si-IL-24), or siRNA against STAT3 (si-STAT3). siRNA transfection efficiently knocked down IL-24 mRNA expression and STAT3 mRNA and protein expression (Supplementary Figure S2C–E). si-control- or si-IL-24-transfected NHEKs were treated with tapinarof (500 nM) for qRT-PCR and Western blotting analyses of FLG and LOR expression. Tapinarof induced the upregulation of FLG and LOR mRNA and protein, which was enhanced by the knockdown of IL-24 expression (Figure 3A–D). In addition, the knockdown of STAT3 expression enhanced the upregulation of FLG and LOR mRNA and protein induced by tapinarof treatment (Figure 3E–H).

Smith et al. [11] reported that tapinarof upregulated the expression of IVL, an essential skin barrier protein that is also decreased in AD [19]. However, in our studies, since IL-24 did not downregulate IVL expression [15], inhibition of the IL-24/STAT3 axis did not enhance the upregulation of IVL expression induced by tapinarof in NHEKs (data not shown).

### 2.4. JAK Inhibitors Augmented Tapinarof-Induced Upregulation of FLG and LOR Expression in NHEKs

**Figure 4:**
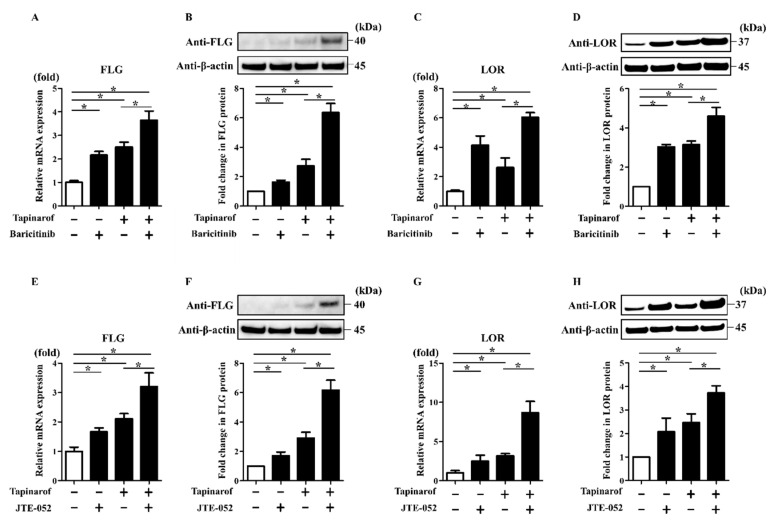
JAK inhibitors augmented tapinarof-induced upregulation of FLG and LOR expression in NHEKs. NHEKs were treated with baricitinib (1 µM) (**A**–**D**) or JTE-052 (1 µM) (**E**–**H**) in the presence or absence of tapinarof (500 nM) for 72 h for qRT-PCR and for 96 h for Western blotting analyses. (**A**,**C**,**E**,**G**) FLG and LOR mRNA expression was assessed by qRT-PCR. Representative data of three independent experiments with similar results are shown. (**B**,**D**,**F**,**H**) FLG and LOR protein expression was analyzed by Western blotting with anti-FLG and anti-LOR antibodies. (**Upper**) Representative blot images of three independent experiments with similar results are shown. (**Lower**) FLG or LOR protein levels are normalized to β-actin protein levels using ImageJ and expressed as fold change over the control group. (**A**–**H**) Error bars represent mean ± standard deviation; *n* = 3 for each group. Statistically significant differences are presented: * *p* < 0.05.

Since JAK inhibitors, such as baricitinib and JTE-052, utilized in the treatment of AD, potentially interfere with the JAK/STAT3 axis [16,17,18], we expected that baricitinib and JTE-052 might enhance the upregulation of FLG and LOR expression induced by tapinarof treatment. To investigate this, NHEKs were treated with DMSO (control), baricitinib (1 µM), or JTE-052 (1 µM) in the presence or absence of tapinarof (500 nM) for qRT-PCR and Western blotting analyses of FLG and LOR. Treatment with baricitinib or JTE-052 alone upregulated FLG and LOR mRNA and protein in NHEKs (Figure 4). In addition, tapinarof induced the upregulation of FLG and LOR mRNA and protein, which was augmented by combined treatment with baricitinib (Figure 4A–D) or JTE-052 (Figure 4E–H). Baricitinib (1 µM) and JTE-052 (1 µM) did not affect cell viability (Supplementary Figure S1).

### 2.5. JAK Inhibitors Enhanced the Reversing Effects of Tapinarof on the FLG and LOR Downregulation Induced by IL-4, a Key Th2 Cytokine of AD

**Figure 5:**
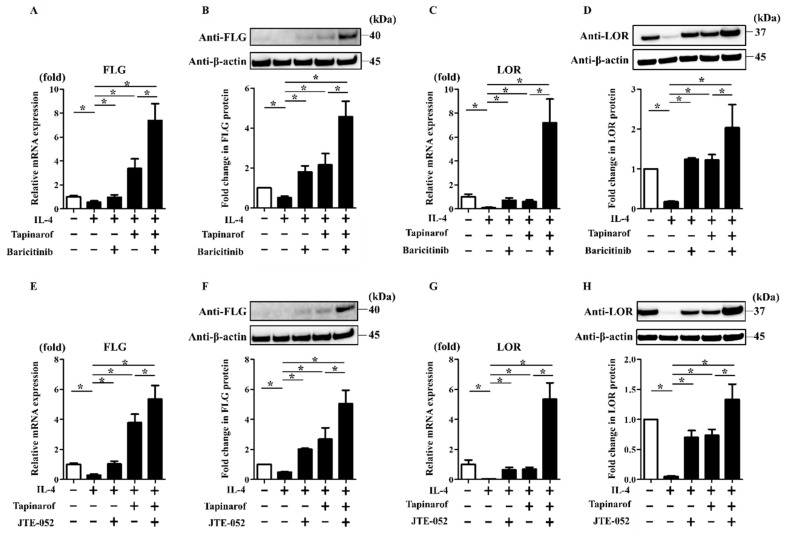
JAK inhibitors enhanced the reversing effects of tapinarof on the FLG and LOR downregulation induced by IL-4. NHEKs were treated with IL-4 (50 ng/mL) in the presence or absence of baricitinib (1 µM) and tapinarof (500 nM) (**A**–**D**) or JTE-052 (1 µM) and tapinarof (500 nM) (**E**–**H**) for 72 h for qRT-PCR and for 96 h for Western blotting analyses. (**A**,**C**,**E,G**) FLG and LOR mRNA expression was assessed by qRT-PCR. Representative data of three independent experiments with similar results are shown. (**B**,**D**,**F**,**H**) FLG and LOR protein expression was analyzed by Western blotting with anti-FLG and anti-LOR antibodies. (**Upper**) Representative blot images of three independent experiments with similar results are shown. (**Lower**) FLG or LOR protein levels are normalized to β-actin protein levels using ImageJ and expressed as fold change over the control group. (**A**–**H**) Error bars represent mean ± standard deviation; *n* = 3 for each group. Statistically significant differences are presented: * *p* < 0.05.

To further examine whether combination treatment with baricitinib or JTE-052 and tapinarof improves the IL-4-induced downregulation of FLG and LOR expression, which partially recapitulates keratinocytes in AD [9], NHEKs were exposed to IL-4 (50 ng/mL) in the presence or absence of baricitinib (1 µM) or JTE-052 (1 µM) and tapinarof (500 nM) for qRT-PCR and Western blotting analyses of FLG and LOR. Treatment with baricitinib or JTE-052 alone restored the IL-4-induced downregulation of FLG and LOR mRNA and protein (Figure 5). In addition, tapinarof treatment alone reversed the IL-4-induced downregulation of FLG and LOR mRNA and protein, which was further restored by treatment with baricitinib (Figure 5A–D) or JTE-052 (Figure 5E–H).

## 3. Discussion

**Figure 6:**
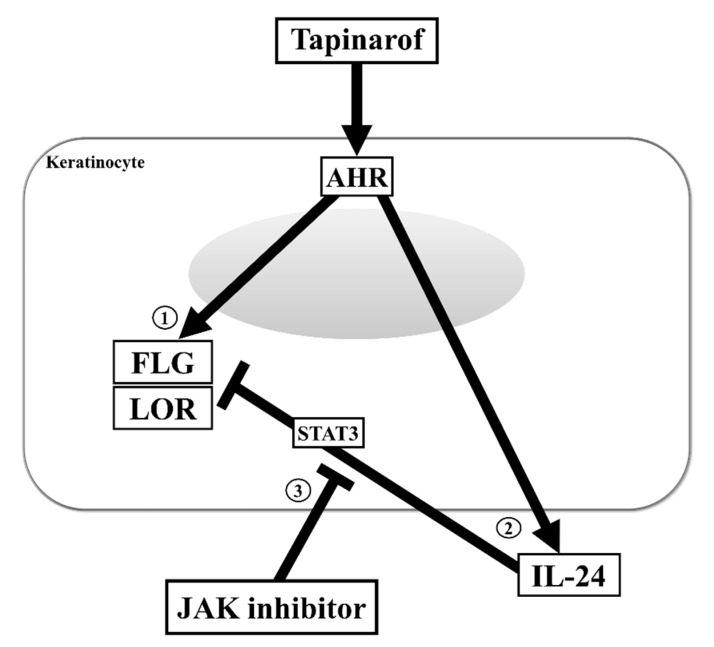
Inhibition of the IL-24/STAT3 axis during AHR activation enhances the upregulation of FLG and LOR expression in keratinocytes. (1) Tapinarof upregulates FLG and LOR expression in an AHR-dependent manner. (2) Tapinarof produces IL-24, an activator of the JAK/STAT3 axis that negatively regulates FLG and LOR expression, via AHR activation. (3) Blockade of the IL-24-STAT3 axis during AHR activation using JAK inhibitor supports the upregulation of FLG and LOR expression induced by tapinarof treatment.

Skin barrier dysfunction and immunological abnormalities are critical in the pathogenesis of AD, in which the reduction in the important epidermal barrier proteins FLG and LOR caused by type 2 cytokine IL-4/13 signaling has been extensively highlighted [2,3,6,7,16]. Recently, topical tapinarof, an AHR modulator, has attracted attention due to its effectiveness in treating AD patients in clinical trials [6,11,12,26]. In this study, we demonstrate that: (1) tapinarof increased FLG and LOR in an AHR-dependent manner and restored the IL-4-mediated FLG and LOR downregulation in human keratinocytes, whereas (2) tapinarof produced IL-24, a negative regulator of FLG and LOR, via AHR activation. In addition, (3) blockade of the IL-24-STAT3 axis during AHR activation using baricitinib or JTE-052, JAK inhibitors, supported the increases of FLG and LOR induced by tapinarof treatment and strengthened the reversing effects of tapinarof on the decreases in FLG and LOR expression upon IL-4 exposure (Figure 6). Given these results, we propose that tapinarof alone ameliorates barrier defects, and its combination with JAK inhibitors amplifies this impact, leading to improvements in barrier dysfunction in AD.

As mentioned above, IL-24 was previously determined to be a target cytokine of some oxidative AHR agonists, such as TCDD, benzo(a)pyrene, particulate matter, and UVB irradiation [20,21,22,23]. The current study reveals for the first time that tapinarof stimulated keratinocytes to generate IL-24 secretion. Smith et al. [11] reported that tapinarof is an antioxidative AHR modulator. Here, we confirmed that tapinarof did not induce reactive oxygen species (ROS) production in NHEKs (Supplementary Figure S3). Although it has been shown that the oxidative or antioxidative type of AHR ligand is an important factor determining the different downstream responses of AHR signaling [6,27], both types induce IL-24 expression. In addition, AHR-binding sequences (GCGTG) are found in the promoter region of the IL-24 gene [23,28]. Furthermore, knockdown of AHR significantly attenuated the upregulation of IL-24 induced by tapinarof (Figure 2H,I). These results indicated that AHR activation plays an important role in the mechanism of tapinarof-mediated IL-24 expression. Nevertheless, AHR knockdown did not completely inhibit the increase in IL-24 expression induced by tapinarof, suggesting another possible mechanism of IL-24 induction. Therefore, further studies are needed to understand this mechanism.

IL-24 signals through its receptor composed of either IL-22R1 or IL-20R1, and IL-20R2, activating JAK-STAT3 signaling cascades that produce downstream effects, including the reduction in FLG and LOR expression [14,15]. We confirmed that tapinarof did not alter IL-20R1 or IL-20R2 expression and upregulated IL-22R1 expression in NHEKs (Supplementary Figure S4A–C) and that abolition of the tapinarof-activated IL-24/STAT3 axis by IL-24 siRNA or STAT3 siRNA amplified the increase in FLG and LOR induced by tapinarof (Figure 3). These results may imply that IL-24 derived from tapinarof-treated keratinocytes could still act through its typical signal transduction.

The level of IL-24 is elevated in the epidermis of AD skin lesions [15,29]; however, the implications of this in the pathophysiology of AD remain poorly explored. To date, the significant reported findings are that type 2 cytokines IL-4-, IL-13-, and IL-31-induced IL-24 pathway causes downregulation of FLG and LOR, which is a mechanism contributing to barrier disruption in AD [15,30,31]. In contrast, despite also upregulating IL-24, tapinarof alone still restores the FLG and LOR downregulation in IL-4-treated NHEKs (Figure 5). There is a possibility that the tapinarof—AHR activation may potently upregulate FLG and LOR expression, in which case tapinarof- and IL-4-induced IL-24 can only partially diminish this impact. Although the mechanisms by which AHR activation promotes terminal differentiation are not fully understood, previous investigations highlighted three pathways that are particularly effective for this: AHR/ARNT/FLG gene promoter, Th2 cytokine–STAT6 axis interference, and OVOL1 nuclear translocation [6,7,9,19,32]. Recently, we asserted that the AHR activation abrogates IL-4-induced IL-33 elevation in NHEKs [27]. Considering that IL-33 was shown to downregulate FLG expression [33], AHR activation may enhance FLG expression by inhibiting IL-4-induced IL-33 upregulation.

In addition to the negative effects on the skin barrier, IL-24 was reported to trigger keratinocytes to secrete IL-8, PGE2, and MMP-1 inflammatory mediators [21], and genetic deficiency in IL-24 was shown to partially prevent mice from developing allergic contact dermatitis after exposure to paraphenylenediamine [34], implying that IL-24 contributes to skin inflammatory responses. These findings indicate that IL-24 plays a detrimental role in inflammatory skin diseases. Therefore, there is a risk that topical tapinarof application may prompt keratinocytes to produce IL-24, resulting in some adverse reactions, including exacerbation of skin inflammation. In this scenario, concomitant JAK inhibitor therapy, which blocks IL-24 signaling, is a favorable option as patients may be able to take advantage of the salubrious effects of tapinarof while avoiding its deleterious ones. Nonetheless, further investigation is required to elucidate the roles of tapinarof-induced IL-24 in AD treatment.

Phototherapy using narrow-band UVB (NB-UVB) irradiation has been utilized in the treatment of AD due to its ability to adequately control the activity of this disease. UVB irradiation therapy has been reported to normalize the expression of barrier proteins in AD patients [35]. Although the precise mechanism behind this has remained largely unknown, FICZ, a photo-product of tryptophan and an endogenous AHR ligand, has been shown to play a crucial role [5,6]. We previously demonstrated that FICZ restored skin barrier dysfunction via AHR in an in vitro AD model [9,10]. Therefore, our results in this study suggest that JAK inhibitors may strengthen the therapeutic effect of NB-UVB against skin barrier dysfunction in AD by blocking the JAK/STAT3 axis activated by FICZ/AHR-induced IL-24. Although no clinical reports on the use of NB-UVB therapy in combination with JAK inhibitors for AD have yet been published, some JAK inhibitors, such as tofacitinib and ruxolitinib, are used with NB-UVB for the treatment of vitiligo [36,37]. However, since NB-UVB therapy may trigger cutaneous carcinogenesis [38], the pathomechanism of which may be modified by JAK inhibitors, more careful observation will be required to perform this therapy.

Topical application of glucocorticoids is the mainstay of AD medication despite their wide range of side effects, including defective epidermal differentiation [18,39,40]. Since it has not been clarified how glucocorticoids affect keratinocyte differentiation induced by AHR activation, we examined whether dexamethasone, a typical glucocorticoid, modifies the tapinarof-induced upregulation of FLG and LOR expression in NHEKs. Dexamethasone treatment did not enhance the upregulation of FLG and LOR mRNA expression induced by tapinarof treatment, although it suppressed tapinarof-induced upregulation of IL-24 mRNA (Supplementary Figure S5B–D). In addition, tapinarof-induced upregulation of the mRNA level of CYP1A1, a typical AHR-responsive gene, was found to be partially inhibited by dexamethasone (Supplementary Figure S5A). These findings suggest that glucocorticoid treatment interferes with AHR activation induced by tapinarof treatment, which is consistent with reports describing that dexamethasone suppresses AHR agonist-mediated CYP1A1 upregulation in human ovarian granulosa [41] and choriocarcinoma cell lines [42]. We also found that baricitinib or JTE-052 did not interfere with the upregulation of CYP1A1 mRNA expression induced by tapinarof treatment (Supplementary Figure S5E,F), confirming that they did not act directly on AHR activation. In addition, baricitinib or JTE-052 did not enhance the upregulation of IL-24 expression induced by tapinarof treatment (Supplementary Figure S5G,H). These findings at least suggest that the combination of tapinarof with JAK inhibitors is more effective than that with glucocorticoids for improving epidermal barrier dysfunction in AD.

In conclusion, we demonstrated that tapinarof alone ameliorates skin barrier dysfunction in vitro. Furthermore, co-treatment of JAK inhibitor with tapinarof augments this benefit by suppressing the IL-24 signaling induced by tapinarof. Our observations provide novel insights into the outcome of combined treatment with AHR modulator and JAK inhibitor, two new agents in development for treating AD, and the associated mechanism. The findings also suggest that this concomitant therapy may be a promising strategy for managing AD.

## 4. Materials and Methods

### 4.1. Reagents and Antibodies

Tapinarof, baricitinib, JTE-052 (MedChemExpress, Monmouth Junction, NJ, USA), and dexamethasone (Sigma-Aldrich, St. Louis, MO, USA) were dissolved in dimethyl sulfoxide (DMSO; Nacalai Tesque, Kyoto, Japan) and stored at −80 °C until used in the experiments. Human recombinant IL-4 and IL-24 were obtained from PeproTech (Rocky Hill, NJ, USA). Anti-human filaggrin monoclonal mouse antibody (Santa Cruz Biotechnology, Dallas, TX, USA), anti-human loricrin monoclonal rabbit antibody (Abcam, Cambridge, UK), anti-AHR monoclonal rabbit antibody, anti-STAT3 monoclonal rabbit antibody, and anti-human β-actin monoclonal mouse antibody (Cell Signaling Technology, Danvers, MA, USA) were used for Western blotting.

### 4.2. Cell Culture

Normal human epidermal keratinocytes (NHEKs) purchased from Lonza (Basel, Switzerland) were grown in serum-free keratinocyte culture medium, namely, KBM^TM^ Gold Basal Medium (Lonza), supplemented with bovine pituitary extract, recombinant epidermal growth factor, insulin, hydrocortisone, transferrin, and epinephrine, at 37 °C in 5% CO_2_. The growth medium was replenished every 2–3 days. Cells reaching confluence (70–90%) were disaggregated with 0.25 mg/mL trypsin/0.01% ethylenediaminetetraacetic acid and then sub-cultured. NHEKs at the second to fourth passage were used for the experiments. Cells were treated with stimulating reagents in KBM^TM^ Gold Basal Medium, supplemented with bovine pituitary extract, insulin, transferrin, and epinephrine. Since FLG and LOR are markers of late differentiation [2,6,7,25,43,44], keratinocytes were allowed to differentiate for 3–5 days in stimulation culture and then utilized in qRT-PCR and Western blotting analyses of FLG and LOR in our experiments. The time course used here is in line with those in several previous studies [25,44,45], but different from those in others [7,9,15,46], which examined these markers at earlier time points. This difference is probably due to FLG and LOR expression depending on the type of stimulating reagent, the target cells [44], and the status of cell confluence [27,43,47].

### 4.3. Cell Viability Determination

The effects of tapinarof, baricitinib, JTE-052, and dexamethasone on NHEK viability were analyzed using the WST-1 assay kit (Takara Bio, Shiga, Japan), in accordance with the manufacturer’s instructions. Optical density was measured on the Beckman Coulter^®^ DTX 800 Multimode Detector (Beckman Coulter, Brea, CA, USA). The results are shown in Supplementary Figure S1.

### 4.4. Transfection of siRNAs against AHR, IL-24, and STAT3

Small interfering RNAs (siRNAs) against AHR (AHR siRNA, s1200), IL-24 (IL-24 siRNA, s21674), and STAT3 (STAT3 siRNA, s743), as well as non-targeting siRNA (control siRNA), were obtained from Ambion (Austin, TX, USA). Cells were incubated in the culture medium with a mixture containing 5 nM siRNA and HiPerFect Transfection reagent (Qiagen, Venlo, The Netherlands) for 48 h and then utilized for further experiments. The knockdown efficiencies of siRNA transfections are presented in Supplementary Figure S2.

### 4.5. Quantitative Reverse-Transcription (qRT)-PCR

Total RNA was extracted using RNeasy^®^ Mini Kit (Qiagen), after which reverse transcription to produce cDNA was conducted using the PrimeScript™ RT reagent kit (Takara Bio). qRT-PCR was performed on a CFX Connect™ Real-time PCR Detection System (Bio-Rad, Hercules, CA, USA). The expression levels of FLG and LOR genes were determined by qRT-PCR using TaqMan™ Fast Advanced Master Mix (Thermo Fisher Scientific, Waltham, MA, USA). Amplification was started as the first step at 50 °C for 2 min and 95 °C for 20 s, followed by 44 cycles of qRT-PCR at 95 °C for 3 s and at 60 °C for 10 s as the second step. mRNA expression was normalized to that of the housekeeping gene YWHAZ. Gene expression levels of IL-24, AHR, CYP1A1, STAT3, IL-20R1, IL-20R2, and IL-22R1 were determined by qRT-PCR utilizing TB Green™ Premix Ex Taq™ (Takara Bio). Amplification was started as the first step at 95 °C for 30 s, followed by 40 cycles of 95 °C for 5 s and 60 °C for 20 s as the second step. mRNA expression was normalized to that of the housekeeping gene β-actin. Experiments were performed in triplicate and repeated three times in separate experiments. Primer sequences are shown in Supplementary Table S1.

### 4.6. Western Blotting Analysis

NHEKs were incubated for 10 min in ice-cold lysis buffer [1 mM ethylene glycol-bis(aminoethyl ether)-tetraacetic acid (EGTA), 2 mM Na_3_VO_4_, 20 mM NaF, 1 mM phenylmethylsulfonylfluoride (PMSF), 1% Triton X-100, RIPA buffer 10× (Nacalai Tesque) consisting of 50 mM Tris-HCl, 150 mM NaCl, 1% NP-40, 0.5% sodium deoxycholate, and protease inhibitor cocktail] (pH 7.3) to isolate protein lysates from cells for the Western blotting analysis of FLG. NHEKs were incubated for 5 min in Complete Lysis-M (Roche Diagnostics, Rotkreuz, Switzerland) for Western blotting analysis of LOR, AHR, and STAT3. The protein concentration of the collected cell lysates was measured using a BCA protein assay kit (Thermo Fisher Scientific), following the manufacturer’s protocol. Equal amounts of protein were mixed with 10% NuPage™ sample reducing agent 10× (Thermo Fisher Scientific) and 25% NuPage™ LDS sample buffer 4× (Thermo Fisher Scientific), followed by heating at 70 °C for 10 min. Next, the proteins were loaded and run on 4–12% Bis-Tris Gel (Thermo Fisher Scientific) at 200 V for 20 min and transferred to PVDF membrane (Merck Millipore, Burlington, MA, USA). The membranes were blocked in WesternBreeze™ blocker/diluent (Thermo Fisher Scientific) for 30 min and then probed with primary antibody overnight at 4 °C. They were subsequently incubated with anti-mouse or anti-rabbit horseradish peroxidase-conjugated IgG secondary antibody (Cell Signaling Technology). Protein bands were visualized with Super Signal West Pico Plus Chemiluminescent Substrate (Thermo Fisher Scientific) using the ChemiDoc Touch Imaging System (Bio-Rad). The band at around 40 kDa was determined to reflect FLG protein expression, as previously reported [48,49]. Experiments were repeated three times in separate experiments.

### 4.7. ELISA

Human IL-24 ELISA Kit (R&D Systems, Minneapolis, MN, USA) was used following the manufacturer’s instructions. Optical density was measured on the Beckman Coulter^®^ DTX 800 Multimode Detector. Experiments were repeated three times in separate experiments.

### 4.8. Detection of ROS Production

Image-iT LIVE Green Reactive Oxygen Species Detection Kit (Thermo Fisher Scientific) was used following the manufacturer’s instructions. In brief, this involved the use of DCFH-DA, a cell-permeable non-fluorescent probe that is de-esterified intracellularly and oxidized to highly fluorescent 2′,7′-dichlorofluorescein in the presence of ROS. NHEKs were treated with tapinarof or benzo(a)pyrene for 6 h, and then washed and incubated with DCFH-DA (25 µM) for 30 min at 37 °C. The fluorescent signal of 2′,7′-dichlorofluorescein, the oxidation product of DCFH-DA, was analyzed using a D-Eclipse confocal laser scanning microscope (Nikon, Tokyo, Japan).

### 4.9. Statistical Analysis

Quantitative data are presented as mean ± standard deviation. The statistical significance of differences between groups was examined by Student’s unpaired two-tailed t-test (for two groups), or one-way analysis of variance (ANOVA), followed by Tukey’s multiple comparison test (multiple groups) using GraphPad PRISM 5.0 software (GraphPad Software, La Jolla, CA, USA). A *p*-value less than 0.05 was considered significant.

## Supplementary Materials

Supplementary materials can be found at https://www.mdpi.com/1422-0067/21/24/9412/s1. Supplementary Figure S1: Cell viability. Supplementary Figure S2: siRNA transfection against AHR, IL-24, or STAT3 downregulated mRNA and protein expression of AHR, IL-24, or STAT3 in NHEKs. Supplementary Figure S3: Effect of tapinarof on intracellular ROS production in NHEKs. Supplementary Figure S4: Tapinarof did not alter IL-20R1 and IL-20R2 mRNA expression and upregulated IL-22R1 mRNA expression in NHEKs. Supplementary Figure S5: Effect of the combination treatment of tapinarof and dexamethasone or JAK inhibitors on mRNA expression of CYP1A1, IL-24, FLG, and LOR, which are genes related to AHR activation, in NHEKs. Supplementary Table S1: Primers for qRT-PCR.

## Abbreviations

AD	atopic dermatitis
AHR	aryl hydrocarbon receptor
FLG	filaggrin
IL-4	interleukin-4
IL-24	interleukin-24
JAK	Janus kinase
LOR	loricrin
NHEK	normal human epidermal keratinocyte
NRF2	nuclear factor erythroid 2-related factor 2
STAT	signal transducer and activator of transcription
TCDD	2,3,7,8-Tetrachlorodibenzo-p-dioxin
Th2	T helper 2 cell
UVB	ultraviolet B

## References

[1] Furue M., Chiba T., Tsuji G., Ulzii D., Kido-Nakahara M., Nakahara T., Kadono T. (2017). Atopic dermatitis: Immune deviation, barrier dysfunction, IgE autoreactivity and new therapies. Allergol. Int..

[2] Kim B.E., Leung D.Y.M. (2018). Significance of skin barrier dysfunction in atopic dermatitis. Allergy Asthma Immunol. Res..

[3] Furue M., Ulzii D., Vu Y.H., Tsuji G., Kido-Nakahara M. (2019). Pathogenesis of atopic dermatitis: Current paradigm. Iran. J. Immunol..

[4] Furue M., Hashimoto-Hachiya A., Tsuji G. (2018). Antioxidative phytochemicals accelerate epidermal terminal differentiation via the AHR-OVOL1 pathway: Implications for atopic dermatitis. Acta Derm. Venereol..

[5] Furue M., Uchi H., Mitoma C., Hashimoto-Hachiya A., Tanaka Y., Ito T., Tsuji G. (2019). Implications of tryptophan photoproduct FICZ in oxidative stress and terminal differentiation of keratinocytes. G. Ital. Dermatol. Venereol..

[6] Furue M., Hashimoto-Hachiya A., Tsuji G. (2019). Aryl hydrocarbon receptor in atopic dermatitis and psoriasis. Int. J. Mol. Sci..

[7] Van Den Bogaard E.H., Bergboer J.G.M., Vonk-Bergers M., Van Vlijmen-Willems I.M.J.J., Hato S.V., Van Der Valk P.G.M., Schröder J.M., Joosten I., Zeeuwen P.L.J.M., Schalkwijk J. (2013). Coal tar induces AHR-dependent skin barrier repair in atopic dermatitis. J. Clin. Investig..

[8] Takei K., Mitoma C., Hashimoto-Hachiya A., Uchi H., Takahara M., Tsuji G., Kido-Nakahara M., Nakahara T., Furue M. (2015). Antioxidant soybean tar Glyteer rescues T-helper-mediated downregulation of filaggrin expression via aryl hydrocarbon receptor. J. Dermatol..

[9] Tsuji G., Hashimoto-Hachiya A., Kiyomatsu-Oda M., Takemura M., Ohno F., Ito T., Morino-Koga S., Mitoma C., Nakahara T., Uchi H. (2017). Aryl hydrocarbon receptor activation restores filaggrin expression via OVOL1 in atopic dermatitis. Cell Death Dis..

[10] Kiyomatsu-Oda M., Uchi H., Morino-Koga S., Furue M. (2018). Protective role of 6-formylindolo[3,2-b]carbazole (FICZ), an endogenous ligand for aryl hydrocarbon receptor, in chronic mite-induced dermatitis. J. Dermatol. Sci..

[11] Smith S.H., Jayawickreme C., Rickard D.J., Nicodeme E., Bui T., Simmons C., Coquery C.M., Neil J., Pryor W.M., Mayhew D. (2017). Tapinarof is a natural AhR agonist that resolves skin inflammation in mice and humans. J. Investig. Dermatol..

[12] Peppers J., Paller A.S., Maeda-Chubachi T., Wu S., Robbins K., Gallagher K., Kraus J.E. (2019). A phase 2, randomized dose-finding study of tapinarof (GSK2894512 cream) for the treatment of atopic dermatitis. J. Am. Acad. Dermatol..

[13] Tsuji G., Takahara M., Uchi H., Matsuda T., Chiba T., Takeuchi S., Yasukawa F., Moroi Y., Furue M. (2012). Identification of ketoconazole as an AhR-Nrf2 activator in cultured human keratinocytes: The basis of its anti-inflammatory effect. J. Investig. Dermatol..

[14] Mitamura Y., Nunomura S., Furue M., Izuhara K. (2020). IL-24: A new player in the pathogenesis of pro-inflammatory and allergic skin diseases. Allergol. Int..

[15] Mitamura Y., Nunomura S., Nanri Y., Ogawa M., Yoshihara T., Masuoka M., Tsuji G., Nakahara T., Hashimoto-Hachiya A., Conway S.J. (2018). The IL-13/periostin/IL-24 pathway causes epidermal barrier dysfunction in allergic skin inflammation. Allergy Eur. J. Allergy Clin. Immunol..

[16] Solimani F., Meier K., Ghoreschi K. (2019). Emerging topical and systemic JAK inhibitors in dermatology. Front. Immunol..

[17] Simpson E.L., Lacour J.P., Spelman L., Galimberti R., Eichenfield L.F., Bissonnette R., King B.A., Thyssen J.P., Silverberg J.I., Bieber T. (2020). Baricitinib in patients with moderate-to-severe atopic dermatitis and inadequate response to topical corticosteroids: Results from two randomized monotherapy phase III trials. Br. J. Dermatol..

[18] Nakagawa H., Nemoto O., Igarashi A., Saeki H., Kaino H., Nagata T. (2020). Delgocitinib ointment, a topical Janus kinase inhibitor, in adult patients with moderate to severe atopic dermatitis: A phase 3, randomized, double-blind, vehicle-controlled study and an open-label, long-term extension study. J. Am. Acad. Dermatol..

[19] Hashimoto-Hachiya A., Tsuji G., Murai M., Yan X., Furue M. (2018). Upregulation of FLG, LOR, and IVL expression by *Rhodiola crenulata* root extract via aryl hydrocarbon receptor: Differential involvement of OVOL1. Int. J. Mol. Sci..

[20] Sun H., Shamy M., Kluz T., Muñoz A.B., Zhong M., Laulicht F., Alghamdi M.A., Khoder M.I., Chen L.C., Costa M. (2012). Gene expression profiling and pathway analysis of human bronchial epithelial cells exposed to airborne particulate matter collected from Saudi Arabia. Toxicol. Appl. Pharmacol..

[21] Jin S.H., Choi D., Chun Y.J., Noh M. (2014). Keratinocyte-derived IL-24 plays a role in the positive feedback regulation of epidermal inflammation in response to environmental and endogenous toxic stressors. Toxicol. Appl. Pharmacol..

[22] Luo Y.H., Kuo Y.C., Tsai M.H., Ho C.C., Tsai H.T., Hsu C.Y., Chen Y.C., Lin P. (2017). Interleukin-24 as a target cytokine of environmental aryl hydrocarbon receptor agonist exposure in the lung. Toxicol. Appl. Pharmacol..

[23] Liu G., Asanoma K., Takao T., Tsukimori K., Uchi H., Furue M., Kato K., Wake N. (2015). Aryl hydrocarbon receptor SNP -130 C/T associates with dioxins susceptibility through regulating its receptor activity and downstream effectors including interleukin 24. Toxicol. Lett..

[24] Kim J.H., Bae H.C., Ko N.Y., Lee S.H., Jeong S.H., Lee H., Ryu W.I., Kye Y.C., Son S.W. (2015). Thymic stromal lymphopoietin downregulates filaggrin expression by signal transducer and activator of transcription 3 (STAT3) and extracellular signal-regulated kinase (ERK) phosphorylation in keratinocytes. J. Allergy Clin. Immunol..

[25] Amano W., Nakajima S., Kunugi H., Numata Y., Kitoh A., Egawa G., Dainichi T., Honda T., Otsuka A., Kimoto Y. (2015). The Janus kinase inhibitor JTE-052 improves skin barrier function through suppressing signal transducer and activator of transcription 3 signaling. J. Allergy Clin. Immunol..

[26] Furue M., Nakahara T. (2020). Revival of AHR agonist for the treatment of atopic dermatitis: Tapinarof. Curr. Treat. Options Allergy.

[27] Tsuji G., Hashimoto-Hachiya A., Yen V.H., Miake S., Takemura M., Mitamura Y., Ito T., Murata M., Furue M., Nakahara T. (2020). Aryl hydrocarbon receptor activation downregulates IL-33 expression in keratinocytes via Ovo-Like 1. J. Clin. Med..

[28] Sun Y.V., Boverhof D.R., Burgoon L.D., Fielden M.R., Zacharewski T.R. (2004). Comparative analysis of dioxin response elements in human, mouse and rat genomic sequences. Nucleic Acids Res..

[29] Bao L., Zhang H., Mohan G.C., Shen K., Chan L.S. (2016). Differential expression of inflammation-related genes in IL-4 transgenic mice before and after the onset of atopic dermatitis skin lesions. Mol Cell Probes..

[30] Clarysse K., Pfaff C.M., Marquardt Y., Huth L., Kortekaas Krohn I., Kluwig D., Lüscher B., Gutermuth J., Baron J. (2019). JAK1/3 inhibition preserves epidermal morphology in full-thickness 3D skin models of atopic dermatitis and psoriasis. J. Eur. Acad. Dermatol. Venereol..

[31] Cornelissen C., Marquardt Y., Czaja K., Wenzel J., Frank J., Lüscher-Firzlaff J., Lüscher B., Baron J.M. (2012). IL-31 regulates differentiation and filaggrin expression in human organotypic skin models. J. Allergy Clin. Immunol..

[32] Sutter C.H., Yin H., Li Y., Mammen J.S., Bodreddigari S., Stevens G., Cole J.A., Sutter T.R. (2009). EGF receptor signaling blocks aryl hydrocarbon receptor-mediated transcription and cell differentiation in human epidermal keratinocytes. Proc. Natl. Acad. Sci. USA.

[33] Imai Y. (2019). Interleukin-33 in atopic dermatitis. J. Dermatol. Sci..

[34] Van Belle A.B., Cochez P.M., de Heusch M., Pointner L., Opsomer R., Raynaud P., Achouri Y., Hendrickx E., Cheou P., Warnier G. (2019). IL-24 contributes to skin inflammation in para-phenylenediamine-induced contact hypersensitivity. Sci. Rep..

[35] Tintle S., Shemer A., Suárez-Fariñas M., Fujita H., Gilleaudeau P., Sullivan-Whalen M., Johnson-Huang L., Chiricozzi A., Cardinale I., Duan S. (2011). Reversal of atopic dermatitis with narrow-band UVB phototherapy and biomarkers for therapeutic response. J. Allergy Clin. Immunol..

[36] Phan K., Phan S., Shumack S., Gupta M. (2020). Repigmentation in vitiligo using janus kinase (JAK) inhibitors with phototherapy: Systematic review and meta-analysis. J. Dermatol. Treat..

[37] Peterson D., King B. (2020). UVL in combination with other therapies for vitiligo: Synergy or necessity?. J. Am. Acad. Dermatol..

[38] Raone B., Patrizi A., Gurioli C., Gazzola A., Ravaioli G.M. (2018). Cutaneous carcinogenic risk evaluation in 375 patients treated with narrowband-UVB phototherapy: A 15-year experience from our institute. Photodermatol. Photoimmunol. Photomed..

[39] Demerjian M., Choi E.H., Man M.Q., Chang S., Elias P.M., Feingold K.R. (2009). Activators of PPARs and LXR decrease the adverse effects of exogenous glucocorticoids on the epidermis. Exp. Dermatol..

[40] Hatano Y., Elias P.M., Crumrine D., Feingold K.R., Katagiri K., Fujiwara S. (2011). Efficacy of combined peroxisome proliferator-activated receptor-α ligand and glucocorticoid therapy in a murine model of atopic dermatitis. J. Investig. Dermatol..

[41] Wang S.H., Liang C.T., Liu Y.W., Huang M.C., Huang S.C., Hong W.F., Su J.G.J. (2009). Crosstalk between activated forms of the aryl hydrocarbon receptor and glucocorticoid receptor. Toxicology.

[42] Stejskalova L., Rulcova A., Vrzal R., Dvorak Z., Pavek P. (2013). Dexamethasone accelerates degradation of aryl hydrocarbon receptor (AHR) and suppresses CYP1A1 induction in placental JEG-3 cell line. Toxicol. Lett..

[43] Hohl D., Lichti U., Breitkreutz D., Steinert P.M., Roop D.R. (1991). Transcription of the human loricrin gene in vitro is induced by calcium and cell density and suppressed by retinoic acid. J. Investig. Dermatol..

[44] Lozza L., Moura-Alves P., Domaszewska T., Lage Crespo C., Streata I., Kreuchwig A., Puyskens A., Bechtle M., Klemm M., Zedler U. (2019). The henna pigment lawsone activates the aryl hydrocarbon receptor and impacts skin homeostasis. Sci. Rep..

[45] Di Z.H., Ma L., Qi R.Q., Sun X.D., Huo W., Zhang L., Lyu Y.N., Hong Y.X., Chen H.D., Gao X.H. (2016). T helper 1 and T helper 2 cytokines differentially modulate expression of filaggrin and its processing proteases in human keratinocytes. Chin. Med. J. (Engl.).

[46] Chamcheu J.C., Esnault S., Adhami V.M., Noll A.L., Banang-Mbeumi S., Roy T., Singh S.S., Huang S., Kousoulas K.G., Mukhtar H. (2019). Fisetin, a 3,7,3’,4’-tetrahydroxyflavone inhibits the PI3K/Akt/mTOR and MAPK pathways and ameliorates psoriasis pathology in 2D and 3D organotypic human inflammatory skin models. Cells.

[47] Lee Y.S., Yuspa S.H., Dlugosz A.A. (1998). Differentiation of cultured human epidermal keratinocytes at high cell densities is mediated by endogenous activation of the protein kinase C signaling pathway. J. Investig. Dermatol..

[48] Jia H.Y., Shi Y., Luo L.F. (2016). Asymmetric stem-cell division ensures sustained keratinocyte hyperproliferation in psoriatic skin lesions. Int. J. Mol. Med..

[49] Gschwandtner M., Mildner M., Mlitz V. (2013). Histamine suppresses epidermal keratinocyte differentiation and impairs skin barrier function in a human skin model. Allergy.

