# Supplementary material for: IL-24 Negatively Regulates Keratinocyte Differentiation Induced by Tapinarof, an Aryl Hydrocarbon Receptor Modulator: Implication in the Treatment of Atopic Dermatitis
Source: Int J Mol Sci. 2020 Dec 10;21(24):9412. doi: 10.3390/ijms21249412 (PMC7764126; doi:10.3390/ijms21249412)
Supplement: Supplementary file 1 [file ijms-21-09412-s001.pdf]

IL-24 negatively regulates keratinocyte differentiation induced by tapinarof, an aryl hydrocarbon receptor modulator: implication in the treatment of atopic dermatitis

Supplementary Materials

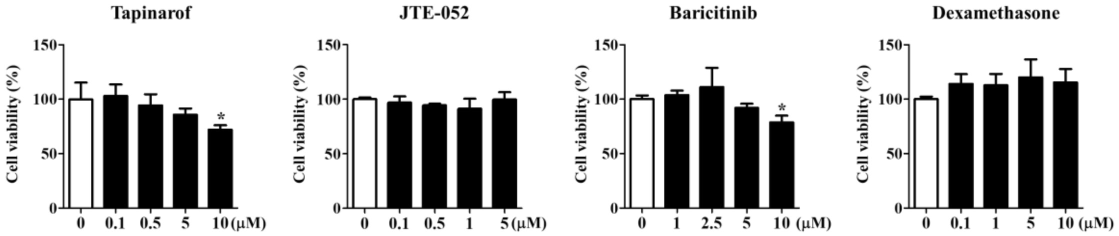

Supplementary Figure S1. Cell viability. NHEKs were treated with tapinarof, JTE-052, baricitinib, or dexamethasone at the stated doses for 24 h. WST-1 cell proliferation assay was performed to check cell viability. Statistically significant differences in expression between the control and NHEKs treated with tapinarof, JTE-052, baricitinib, or dexamethasone are presented: *P < 0.05.

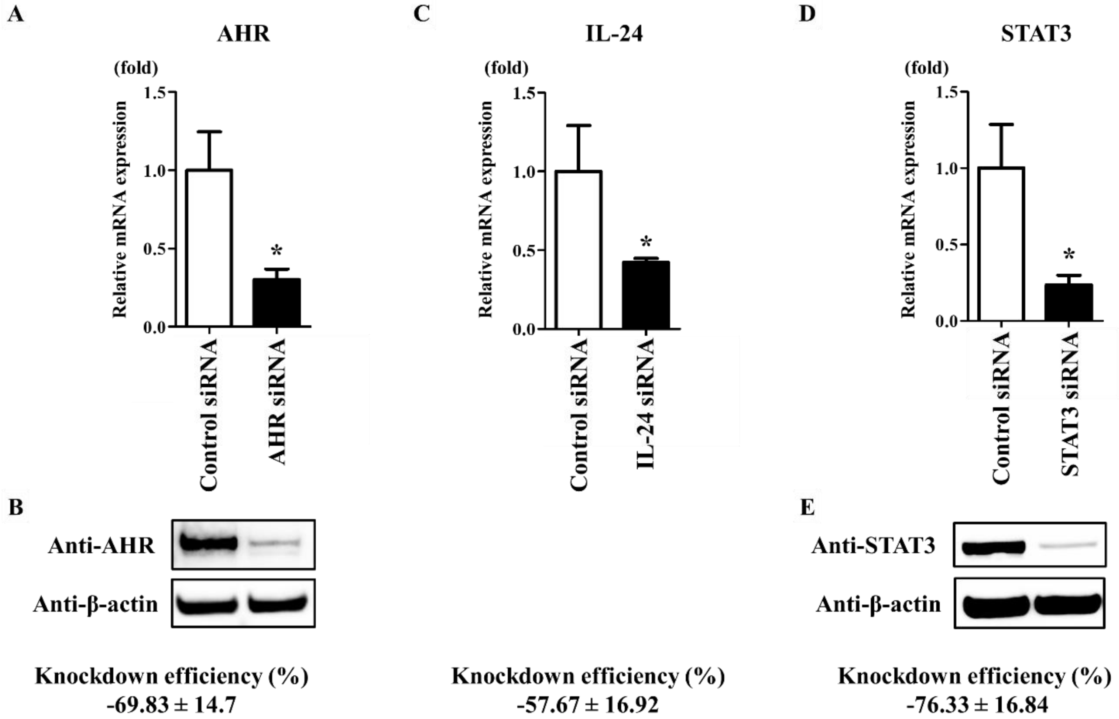

Supplementary Figure S2. siRNA transfection against AHR, IL-24, or STAT3 downregulated mRNA and protein expression of AHR, IL-24, or STAT3 in NHEKs. NHEKs were transfected with control siRNA (si-control), siRNA against AHR (si-AHR), siRNA against IL-24 (si-IL-24), or siRNA against STAT3 (si-STAT3). **(A,C,D)** AHR, IL-24, or STAT3 mRNA expression was assessed by qRT-PCR. Error bars represent mean ± standard deviation; n = 3 for each group. Statistically significant differences in expression between NHEKs transfected with control siRNA and NHEKs treated with AHR siRNA, IL-24 siRNA, or STAT3 siRNA are presented: *P < 0.05. **(B,E)** AHR or STAT3 protein expression was analyzed by Western blotting with anti-AHR or anti-STAT3 antibodies.

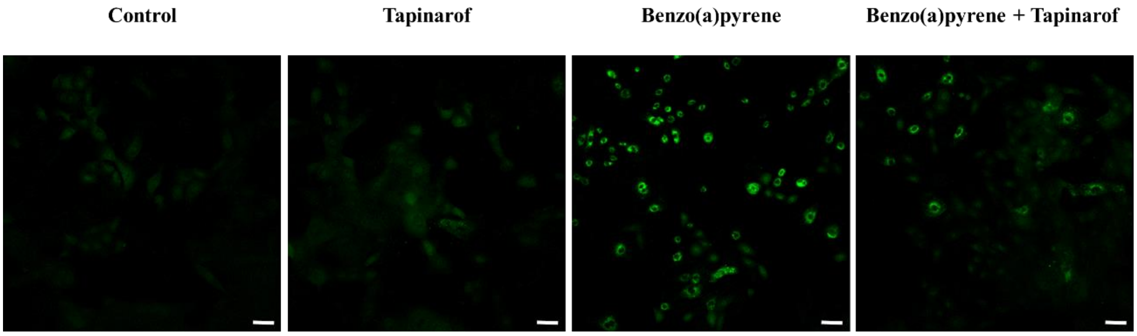

Supplementary Figure S3. Effect of tapinarof on ROS production in NHEKs. NHEKs were treated with DMSO (control), tapinarof (500 nM), or benzo(a)pyrene (1 μM) for 6 h. ROS production was evaluated using the fluorescence signal of 2′,7′-dichlorofluorescein visualized by confocal laser scanning microscopy. Scale bar = 20 μm. Compared with DMSO (negative control), tapinarof alone did not induce ROS production in NHEKs. In contrast, the potent ROS inducer benzo(a)pyrene (positive control) clearly induced production in NHEKs, which was attenuated by combined treatment with tapinarof.

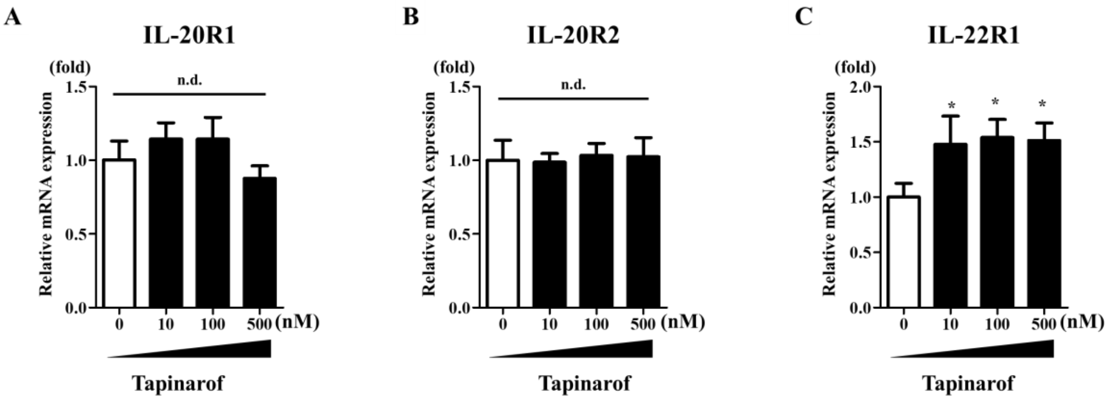

Supplementary Figure S4. Effect of tapinarof on IL-24 receptor mRNA expression in NHEKs. NHEKs were treated with tapinarof (500 nM) for 24 h. IL-20R1, IL-20R2, or IL-22R1 mRNA expression was assessed by qRT-PCR. Error bars represent mean ± standard deviation; n = 3 for each group. n.d. no difference. Statistically significant differences in expression between the control and NHEKs treated with tapinarof are presented: *P < 0.05.

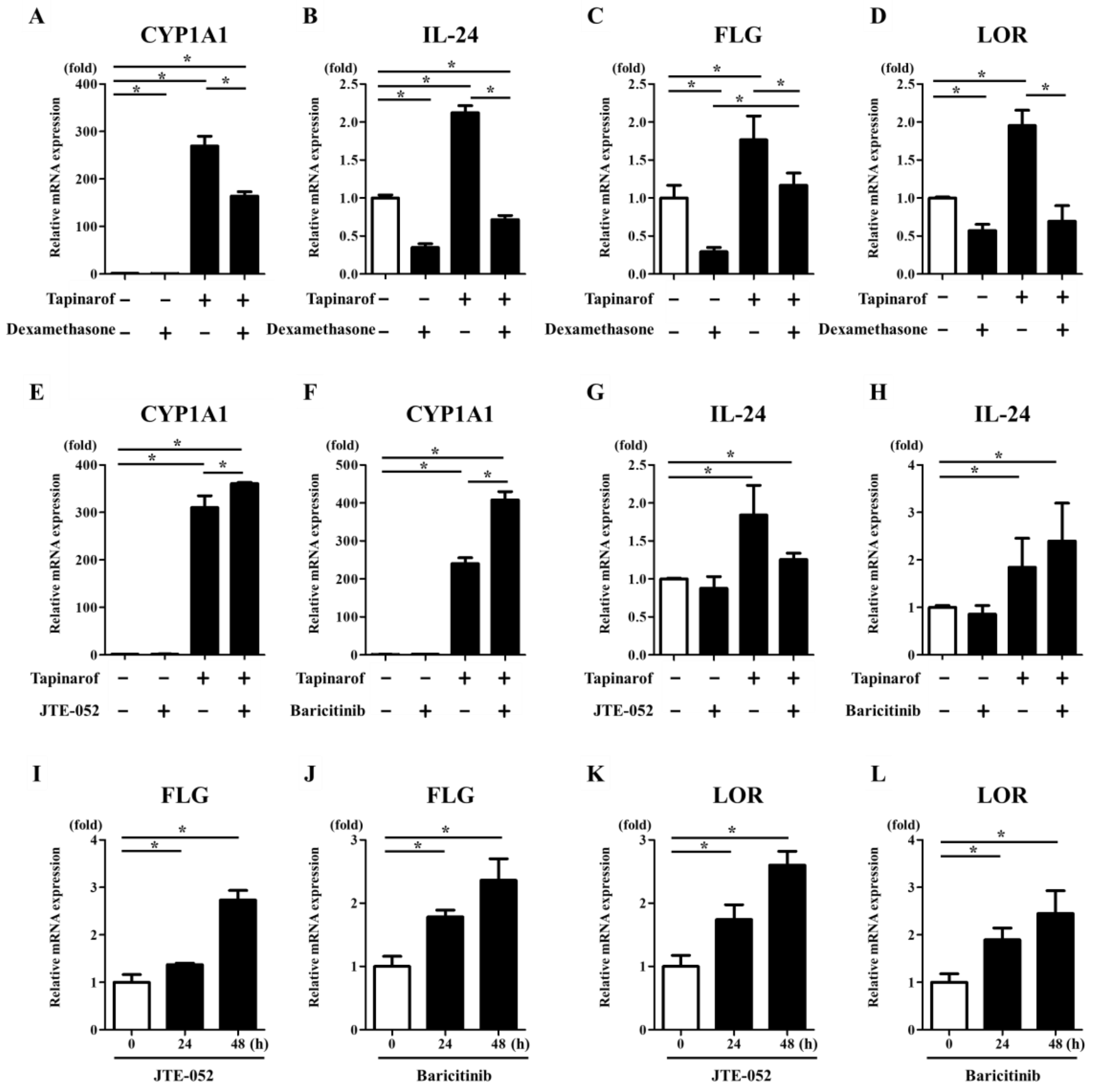

Supplementary Figure S5. Effect of combination treatment with tapinarof and dexamethasone or JAK inhibitors on the mRNA expression of CYP1A1, IL-24, FLG, and LOR, which are genes related to AHR activation, in NHEKs. NHEKs were treated with dexamethasone (1 μM) **(A–D)**, JTE-052 (1 μM) **(E,G,I,K),** or baricitinib (1 μM) **(F,H,J,L)** in the presence or absence of tapinarof (500 nM). The mRNA expression of CYP1A1 **(A,E,F)**, IL-24 **(B,G,H)**, FLG **(C,I,J)**, and LOR **(D,K,L)** was assessed by qRT-PCR. Error bars represent mean ± standard deviation; n = 3 for each group. Statistically significant differences are presented: *P < 0.05.

Supplementary Table S1. Primers for qRT-PCR.

Gene	Forward primer	Reverse primer
AHR	5′-ATCACCTACGCCAGTCGCAAG-3′	5′-AGGCTAGCCAAACGGTCCAAC-3′
IL24	5′-CCAGGAGGAACACGAGACTGA-3′	5′-CAGAAGGGTCTGGCTAAAGTCCAC-3′
STAT3	5′-TCCTGGCCCTTTGGAACGAA -3′	5′-GGATCTGGGTCTTACCGCTGA -3′
IL20R1	5′-AACAGAACGTGGTCCCAGTG -3′	5′-TTCTCAGAAGGCTGAGCACG -3′
IL20R2	5′-ATGCAGACTTTCACAATGGTTCTA -3′	5′-ATGGCCACTTCATCTGTGAGC -3′
IL22R1	5′-CTCTGCAGCACACTACCCTC -3′	5′-ATGTCTTCCAGGGTTAGCCG-3′
β-actin	5′-ATTGCCGACAGGATGCAGA-3′	5′-GAGTACTTGCGCTCAGGAGGA-3′
CYP1A1	PPH01271E (SABiosciences)	
FLG	Hs00856927_g1 (TaqMan™ Gene Expression Assay)	
LOR	Hs01894962_s1 (TaqMan™ Gene Expression Assay)	
YWHAZ	Hs01122445_g1 (TaqMan™ Gene Expression Assay)	
